# Spring constant of a tuning-fork sensor for dynamic force microscopy

**DOI:** 10.3762/bjnano.3.90

**Published:** 2012-11-29

**Authors:** Dennis van Vörden, Manfred Lange, Merlin Schmuck, Nico Schmidt, Rolf Möller

**Affiliations:** 1 Faculty of Physics, University of Duisburg-Essen, Lotharstr. 1–21 47048 Duisburg, Germany

**Keywords:** atomic force microscopy, finite element method, spring constant, thermal fluctuation, tuning fork

## Abstract

We present an overview of experimental and numerical methods to determine the spring constant of a quartz tuning fork in qPlus configuration. The simple calculation for a rectangular cantilever is compared to the values obtained by the analysis of the thermal excitation and by the direct mechanical measurement of the force versus displacement. To elucidate the difference, numerical simulations were performed taking account of the real geometry including the glue that is used to mount the tuning fork.

## Introduction

Quartz tuning forks provide excellent self-sensing probes in scanning probe microscopy, offering several advantages compared to the standard microfabricated silicon-based cantilevers [1–2]. Frequency-modulation atomic force microscopy (FM-AFM) with a tuning-fork sensor has had a major impact on fundamental and scientific research, e.g., by resolving the structure of a molecule [3] or even determining the structure of an unknown organic molecule [4].

In FM-AFM, the motion of the sensor is given in very good approximation by a harmonic oscillator. For the limit of small amplitudes the measurement of the frequency shift provides the average force gradient caused by the interaction between the tip and sample surface, according to

[1]
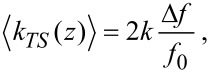


where 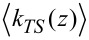 is the average force gradient between tip and sample, Δ*f* is the frequency shift, *k* is the spring constant of the sensor and *f*_0_ is the resonance frequency of the sensor without interaction with the sample.

While the resonance frequency may be measured accurately in the experiment, it is more difficult to evaluate the spring constant *k*. However, the latter is required to evaluate the force gradient and other physical quantities, e.g., the energy dissipated due to the interaction between tip and sample.

For microfabricated cantilevers several methods to evaluate *k* have been demonstrated. The most obvious, although technically difficult, method is to measure the static deflection as a function of the applied force [5–11]. If an additional mass is attached to the cantilever the spring constant can be determined by the change of the resonance frequency [12–14]. By measuring the amplitude of the thermal noise, *k* can be evaluated in situ, e.g., in a vacuum system prior to the measurement, without any modification of the experimental arrangement [15–20]. However, this requires a good signal-to-noise ratio for the measurement of the beam deflection.

For the analysis of data obtained by tuning-fork sensors *k* is often simply calculated based on the formula for a cantilever beam [21]. In principal the experimental techniques mentioned above can be applied for tuning forks sensors as well [22–24].

In the present paper we compare the results for the determination of the spring constant of tuning fork sensors in the qPlus configuration [1–2] based on the following methods: a simple calculation for a cantilever beam; the measured deflection as a function of the applied force; the thermal noise; and a numerical simulation by the finite-element method.

## Result and Discussion

### Calculation for a rectangular beam

The formula for the spring constant of a beam that is clamped on one side is

[2]
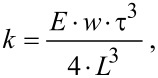


where *E* is the Young’s modulus (for quartz), τ is the thickness, *w* the width, and *L* the length of a prong. For the cantilevers used in our experiments, *E* = 78.7 GPa, *t* = 0.41 ± 0.003 mm, *w* = 0.24 ± 0.003 mm, and *L* = 2.94 ± 0.003 mm, yielding a value of

[3]
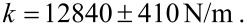


### Experimental evaluation of the spring constant

#### Beam deflection as function of applied force

To a good approximation, the force *F* exerted by the tuning fork (TF) in the qPlus configuration is given by Hooke’s law *F* = −*kz*, with the spring constant *k* and the deflection *z*.

**Figure 1:**
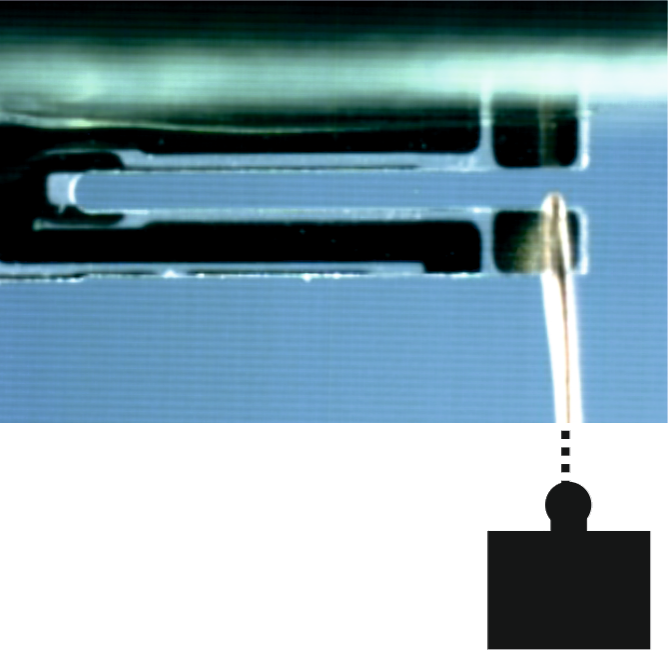
Setup for evaluating the spring constant by Hooke’s law. The deflection is measured by analyzing differences between images of the loaded and unloaded TF using WSxM [26].

For the experiment, the TFs are glued to a holder in exactly the same way as for the low-temperature noncontact AFM developed in our group [25]. To apply a force on the TF a loop is formed by a thin wire, which is hooked as far as possible to the end of the free prong (Figure 1). Two weights with masses of *m*_1_ = 14.5 g and *m*_2_ = 19.3 g are used to apply the force. The measurements are performed at room temperature. The runs of loading and unloading are repeated three times for each TF. The deflection is monitored by a CCD-chip (Sony ICD098BQ Color: 640 × 480, 4.5 mm diagonal, 5.6 μm × 5.6 μm) and an objective lens (Cosmicar Pentax TV lens, *f* = 16 mm, 1:1.4) providing an optical resolution of about 7 μm. The images are calibrated by using a scale. By comparing the images for the loaded and unloaded TF, the deflection at the end of the prong is determined, using WSxM [26] and Corel Draw X5 (Corel Corporation, Ottawa, Canada).

**Figure 2:**
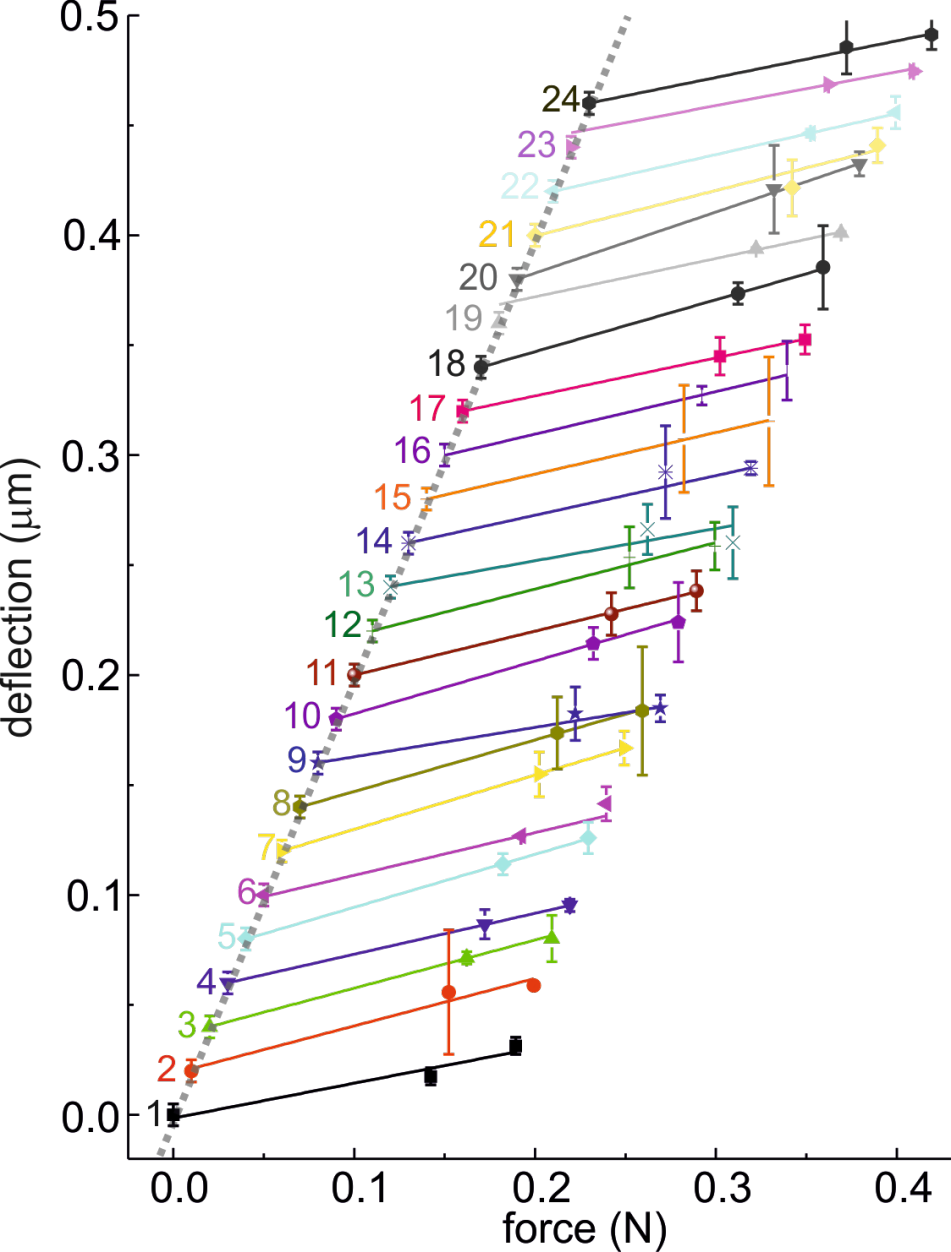
Deflection versus force for 24 TFs using Torr Seal. The individual lines are displaced for clarity.

Two different kind of glues were used. The results for a set of 24 TFs glued to the holder by Torr Seal^®^ (ThorLabs GmbH, Dachau, Germany) are plotted in Figure 2. Similarly a set of 16 TFs glued by UHU plus endfest 300^®^ (UHU GmbH, Bühl, Germany) was measured. Both glues were prepared accurately, according to the corresponding recipe. Care was taken to use approximately the same amount of glue. The results of an analysis of both data sets are given in Table 1.

**Table 1:** Spring constants measured by Hooke’s law. *k* refers to the position at which the load is applied, while *k*_end_ is obtained by extrapolation for the end of the free prong.

	*k* (N/m)	*k*_end_ (N/m)

Torr Seal	9280 ± 960	8190 ± 960
UHU endfest	7500 ± 1520	6590 ± 1520

The results are valid for a point of application of the force that is about 0.3 mm from the end of the prong. According to the numerical calculations by the method of finite elements presented in the following, these values can be extrapolated for the end of the prong by a reduction of 1090 and 910 N/m for Torr Seal and UHU endfest, respectively. The numbers are given in the right column. The values of the spring constants for both glues are significantly smaller than the one calculated for the rectangular beam. The rather large difference between the two types of glues clearly demonstrates that, at least at room temperature, the glue has a major impact on the effective spring constant, although the thickness of the layer of glue is only about 0.04–0.06 mm.

#### Amplitude of the thermal fluctuations

According to the equipartition theorem a thermal energy of ½*k*_B_*T* will be dissipated in every energetically accessible degree of freedom of a system [27]. The tuning fork in the qPlus configuration has one degree of freedom:

[4]
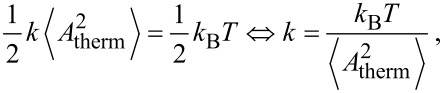


where *k* is the spring constant, *k*_B_ is the Boltzmann’s constant, *T* is the temperature in Kelvin and *A*_therm_ the amplitude of thermal deflection of one prong.

**Figure 3:**
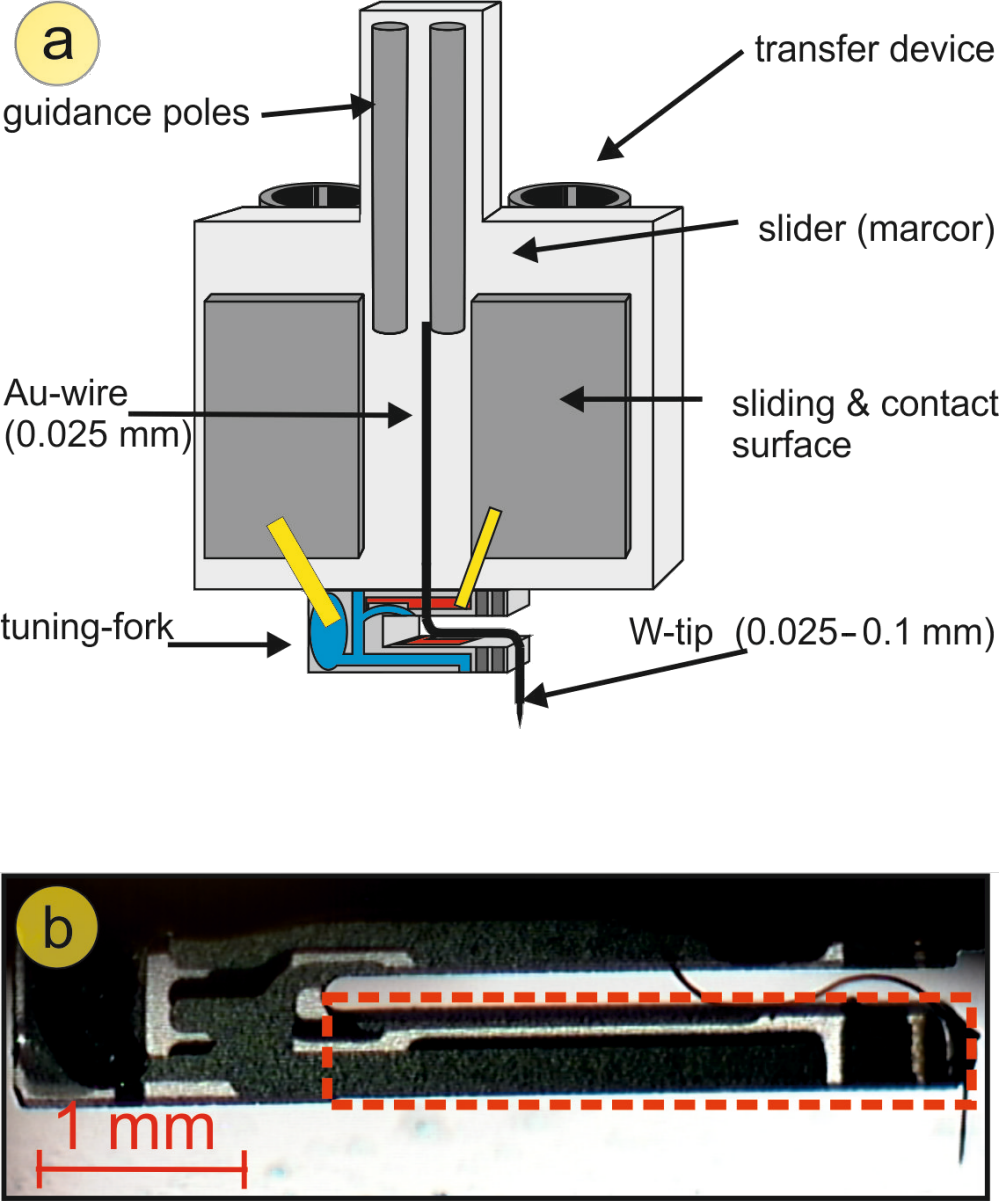
(a) Sketch of the qPlus configuration in our setup. (b) Picture of a mounted tuning fork. The area marked in red indicates the part of the tuning fork that is used to estimate the spring constant based on the formula for a rectangular beam.

The experiments were performed in situ by using a home-built low-temperature tuning-fork AFM (LT-TF-AFM) [25]. Figure 3a shows the carrier onto which the TFs are mounted in the qPlus configuration, i.e., the top side of one prong is glued to a relatively massive part. Based upon initial results for all further experiments Torr Seal was used as the glue. The evaluation of the spring constant was performed for a TF with a tip (e.g., a 25–100 μm tungsten wire) attached to the front face of the free prong of the tuning fork. It is connected separately through a metallic wire (e.g., a 25 μm gold wire) to collect the tunneling current, avoiding crosstalk with the frequency-shift signal.

**Figure 4:**
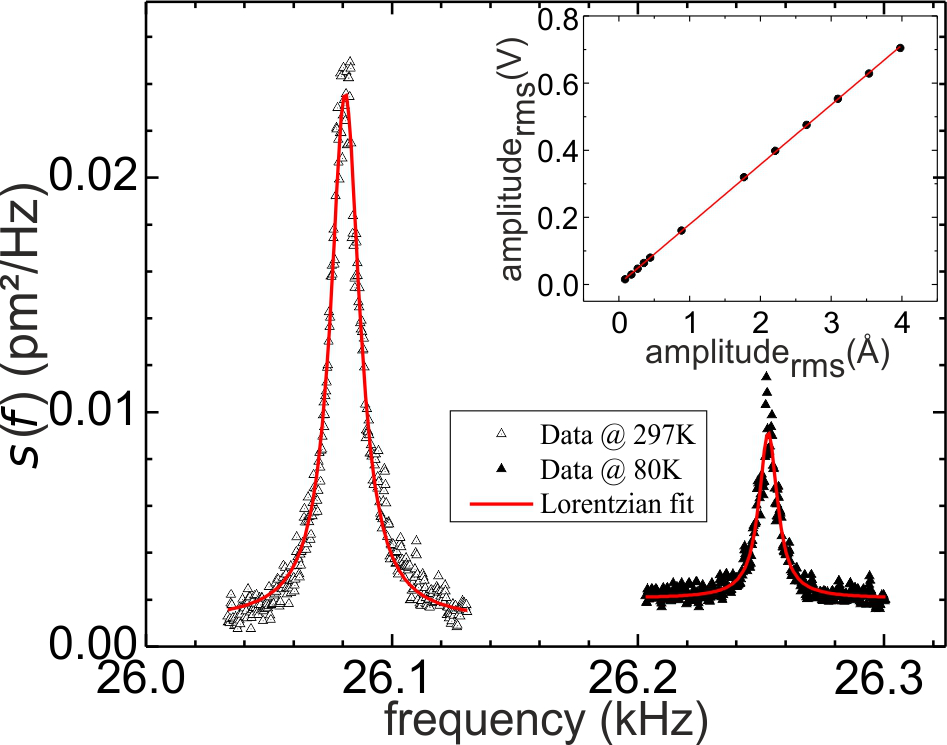
Power spectral density of the thermal fluctuations of a tuning fork at 300 and 80 K. The inset shows the data used to calibrate the sensitivity of the tuning fork.

Prior to the measurements, the sensitivity of the TF in millivolts per nanometer (mV/nm) was calibrated, including the electronics for detection. This is done in several steps. First, the *z*-piezo of the scanning unit is calibrated by measuring the topography of a surface (Cu(111), Ag(111) and Si(111)) with atomic steps of well-known height. Next, the amplitude of the TFs oscillation is varied, while simultaneous monitoring the change in height at constant tunneling current, and the electric signal is detected by the TF. The inset in Figure 4 displays the corresponding graph. This was performed separately at room temperature and at 80 K. Figure 4 shows the power spectral density *s*(*f*) of the thermal fluctuations in picometers squared per hertz (pm^2^/Hz) of a TF at room temperature and at 80 K measured by a FFT-Analyzer (SR 760). A close inspection reveals that not only is the amplitude of the fluctuation lower at 80 K, but also the width is reduced, and the resonance frequency is slightly shifted. To evaluate the amplitude of the thermal fluctuations a Lorentzian was fitted to the spectra:

[5]
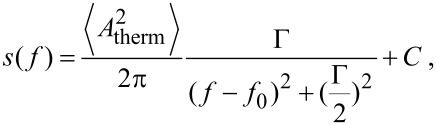


where Γ is the width, *f* is the frequency, *f*_0_ is the resonance frequency and *C* is the offset.

According to Equation 4 the spring constants listed in Table 2 are obtained.

**Table 2:** Spring constants measured by thermal fluctuations. All measurements were performed with Torr Seal as the glue.

*T* (K)	*k* (N/m)

300	8000 ± 500
80	11200 ± 500

The higher spring constant at 80 K is most probably due to the increase in Young’s modulus of the glue. The value at room temperature is rather close to the value obtained by the measurements using Hooke’s law.

### Numerical simulation using the finite element method

To get more insight into the relevant details of the qPlus configuration, numerical calculations by using the method of finite elements were performed. In contrast to the experiment this enables analysis of the influence of one specific parameter, e.g., the thickness of the glue, keeping all the others exactly the same. The simulations were performed with the commercial FEM-software COMSOL Multiphysics 3.5 (COMSOL Multiphysics GmbH, Berlin, Germany) and the additional module “Structural Mechanics”. The model introduced below is simulated by the 3-D model “Solid, Stress-Strain” and statically analyzed by the module “Structural Mechanics”.

**Figure 5:**
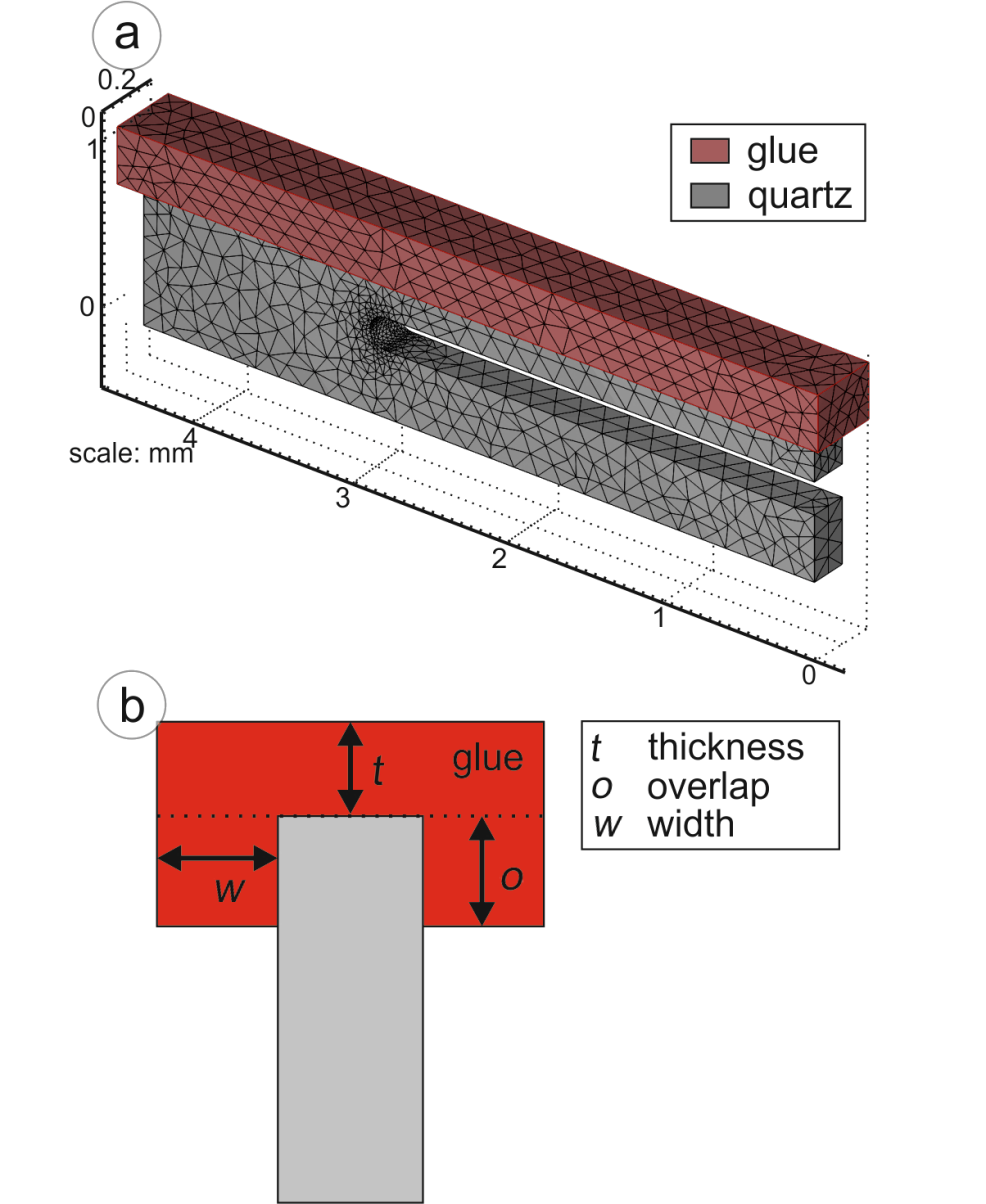
(a) Mesh-grid model of the tuning fork used for FEM simulations. (b) Sketch of the glue enclosing the tuning fork.

A grid model is built for the TF (see Figure 5a) including the glue that is used to attach it to the rigid support. To simulate the experimental configuration as well as possible, it is assumed that the prong of the TF is partially embedded in the glue. Several microscopic images were taken to analyze the experimental geometry in detail. Figure 5b sketches the configuration and the parameters. The thickness is *t* = 0.04 mm, the overlap *o* = 0.15 mm, and the additional width at both sides *w* = 0.1 mm. The given data are valid for the average bonding.

**Figure 6:**
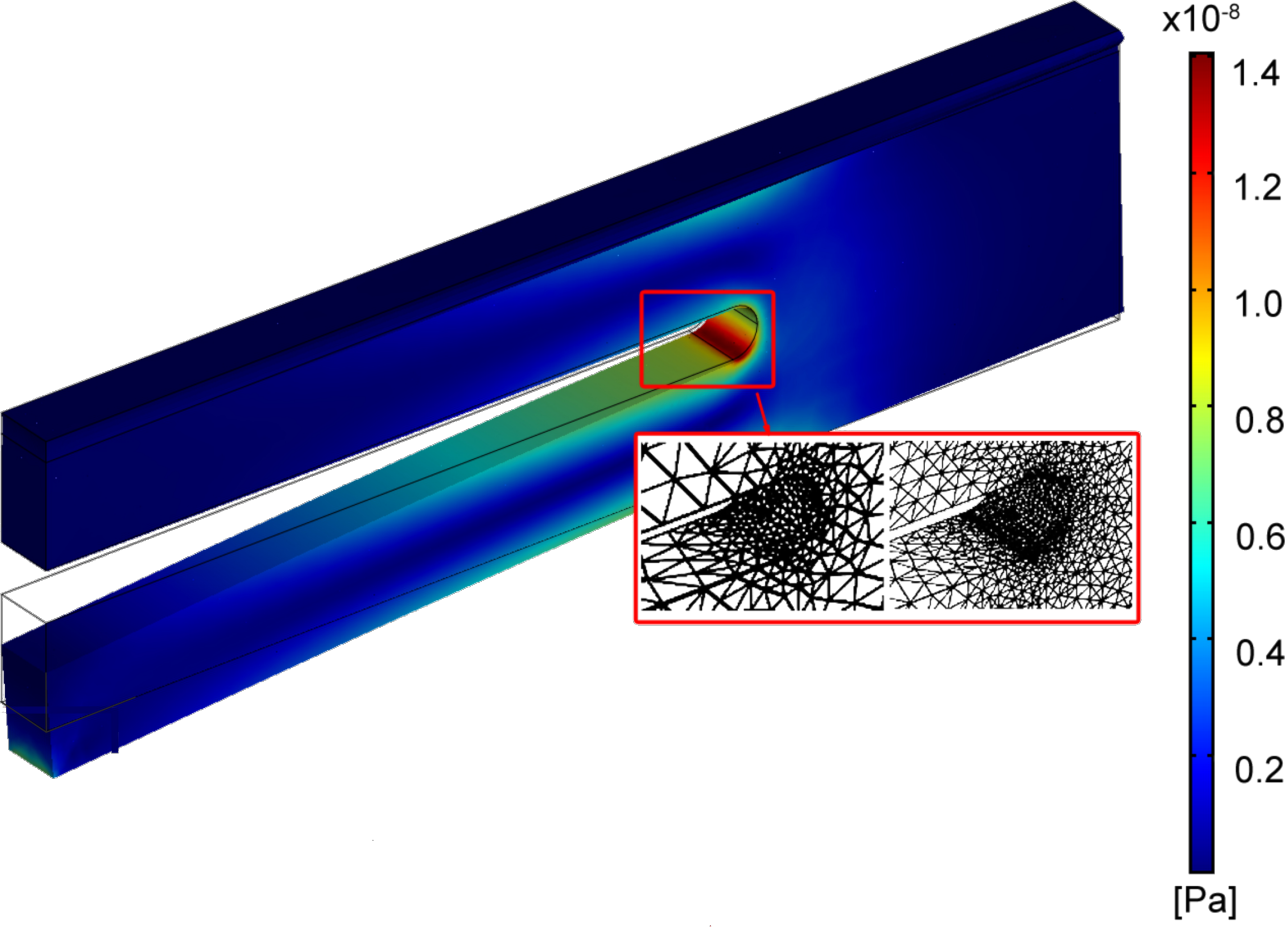
FEM simulation of a deflected TF. The stress is given by the color scale. The inset (red box) shows the refinement of the mesh in the stressed region.

In the simulation, both materials, the glue and the tuning fork (quartz), are considered isotropic. COMSOL Multiphysics needs three different material-specific parameters: Young’s modulus, the Poisson ratio and mass density. The values and the origin of these parameters can be seen in Table 3. As a boundary condition, the interface between the support and the glue is fixed in all directions. The deformation of the TF is calculated for a force applied in the *z*-direction at the end of the free prong. In an iterative procedure, a closer mesh is generated in areas of high deformation after each step, until changes between two sequential steps are marginal. In detail, adaptive mesh refinement was performed by using quadratic Lagrange elements and h-refinement. Figure 6 shows a typical result of the FEM simulation. The mesh is refined in regions of higher stress, e.g., at the link between both prongs.

**Table 3:** Material constants used for FEM simulations. The quartz parameters are taken from COMSOL’s material library, except the Young’s modulus, which was taken from [21]. The glue parameters are taken from [28–30], while the Young’s modulus thereof was determined by converting the Shore D strength using [31].

Material constant	SiO_2_	Torr Seal	UHU endfest

Shore D hardness	—	80	70
Young’s modulus (GPa)	78.7	9.39	5.54
Poisson ratio	0.17	0.45	0.35
mass density (kg/m^3^)	2200	1600	1054

At first a calculation without glue (zero thickness, overlap and width) was performed leading to a spring constant of *k* = 10100 N/m. As to be expected, this value is lower by 19% than for the rectangular beam, because the latter neglects the contribution of the area of the TF linking the two prongs.

**Figure 7:**
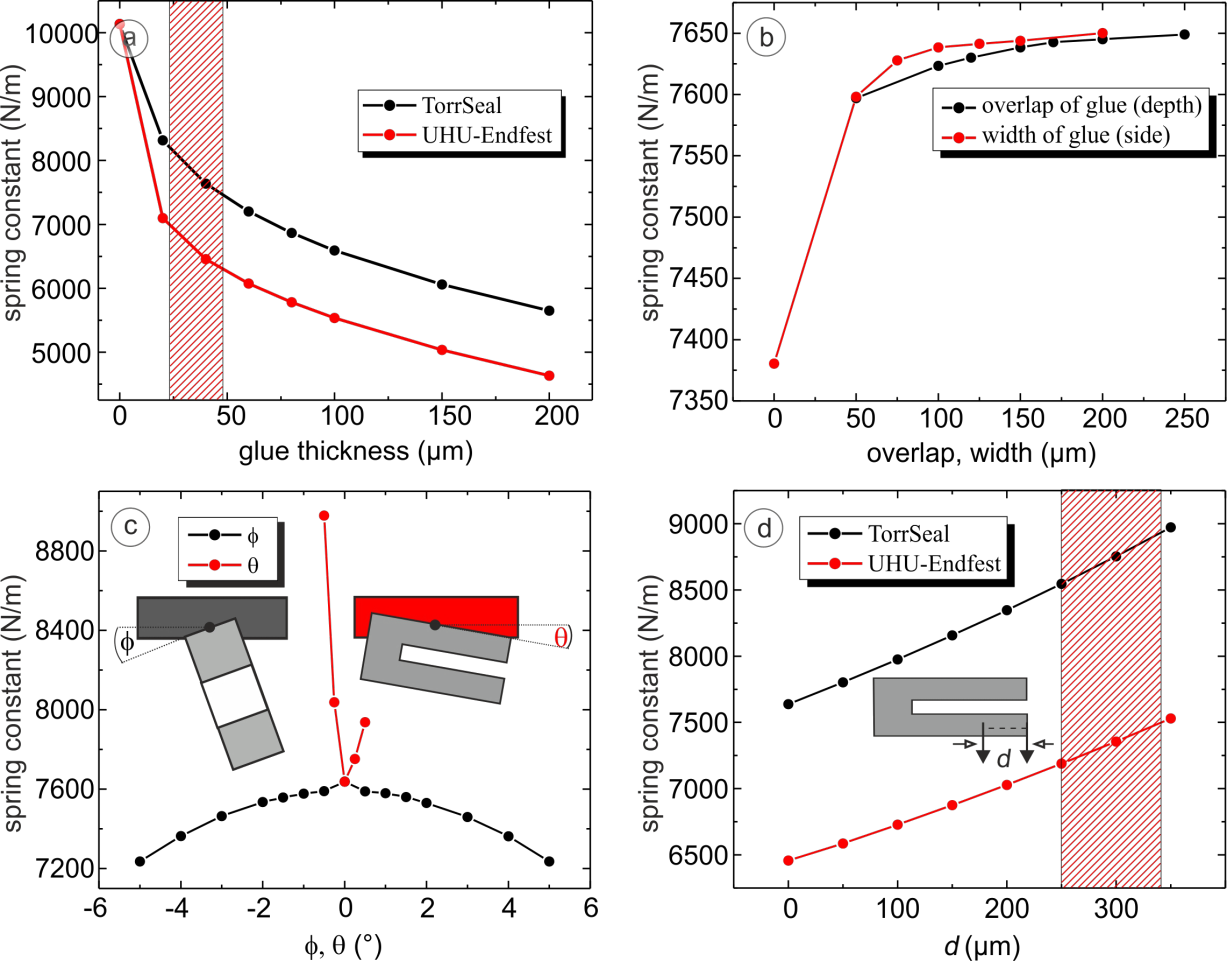
Results of the FEM simulations, when one parameter is varied. The standard parameters are thickness *t* = 0.04 mm, overlap *o* = 0.15 mm, width *w* = 0.1 mm, Φ = 0°, and θ = 0°. The parameters that are not varied are set to the standard values. (a) Spring constant with Torr Seal (black) or UHU endfest (red) as a function of the glue thickness. The hatched area marks the experimental range. (b) Spring constant as a function of the overlap and width of the glue enclosing the upper prong of the tuning fork. (c) Spring constant versus Φ (red) and versus θ (black). While the spring constant decreases symmetrically as a function of Φ, it is asymmetric as a function of θ. (d) Spring constant as a function of the point at which the force was applied for Torr Seal (black) and UHU endfest (red). The shaded area displays the experimental range.

To study the significance of the different parameters, they were varied one by one while keeping the standard values for the remaining ones. Figure 7a displays the calculated effect of a layer of glue between the prong of the TF and the support. As expected, the spring constant decreases with increasing thickness of the glue. Due to the higher Young’s modulus of Torr Seal the reduction is less than for UHU endfest. For the thickness of about 0.04 mm estimated for the experimental setup by microscopic inspection, values for the spring constant for both glues were obtained that are lower than the experimental ones. The influence of the overlap and the width of the overlapping glue is displayed in Figure 7c. The spring constant increases with the width of the overlapping layer of glue up to about 0.05 mm; a further increase of the width has no significant influence. Similarly, the spring constant increases with increasing overlap.

In practice, it is rather difficult to perfectly align the TF with the support. The effect of a tilt is shown in Figure 7b. Tilting around an axis parallel to the long side of the prong results in a decrease of the spring constant that is symmetric with angle. Tilting around an axis that is perpendicular to the TF, as indicated in the figure, leads to an increase of the spring constant that is not symmetric, since either the joint or the end of the TF approaches the support.

For the experimental evaluation of the spring constant using Hooke’s law, the force could not by applied at the very end of the prong. As can be seen in Figure 1, the wire is about 0.2 mm from the end. This leads to an increase of the observed spring constant since the relevant part of the prong is shorter. The resulting difference has been calculated and is displayed in Figure 7d. If the point of application of the force is at a distance of about 0.3 mm from the end, as marked by the hatched area in the figure, the measured spring constant should be about 1000 N/m higher. Based on the FEM calculation (see Figure 7d), the *k* value at the end of the prong can be estimated by extrapolating. This leads to a reduction of the spring constant of about 1090 N/m. The calculated values agree rather well with the ones observed in the experiment for both types of glue.

## Conclusion

The comparison of the different methods to evaluate the spring constant of a TF in the qPlus configuration reveals the importance of the details of the way in which the TF is mounted. Table 4 summarizes the results. An estimate for the spring constant may be obtained by the formula for a rectangular beam with the dimensions of the free prong of the TF. For the TFs used in the present experiments this yields *k* = 12840 N/m. However, this assumes that the one end of the TF is ideally clamped. Since the influence of the part of the TF between the prongs and the attachment to a rigid support is neglected, this value represents an upper limit. When evaluating the spring constant experimentally by measuring the deflection as a function of the applied force, the point of application has to be considered carefully. In the present experiment this was at a distance of 0.2–0.3 mm from the free end, which, according to the numerical simulations presented, leads to an increase of the observed spring constant by about 1000 N/m. The same is true if the tip of the AFM is not mounted at the very end of the prong of the TF. The values listed in Table 4 are calculated by extrapolating the measured values to the very end of the prong.

**Table 4:** Comparison of the different evaluation methods.

Method	*T* (K)	*k* (N/m)	
		UHU endfest	Torr Seal

rectangular beam (no glue)	300	12840 ± 410	12840 ± 410
Hooke’s law	300	6590 ± 1520	8190 ± 960
thermal fluctuation	300		8080 ± 300
thermal fluctuation	80		10950 ± 500
numerical calculation	300	6490	7650
numerical calculation with *t* = 0.0 mm	300	10136	10136

While this method could only be applied ex situ, the spring constant may be evaluated in situ by the amplitude of the thermal fluctuations. Use of a low-temperature setup enables us to analyze the influence of the temperature on the spring constant. In Table 4 the values for 300 and 80 K are given.

The values of the spring constant for both experimental techniques agree well within the experimental errors. As expected these values are lower than for the ideal beam. To analyze the cause of the difference in detail, the method of finite elements (FEM) was applied. It reveals that the value of the rectangular beam of 12450 N/m is reduced to 10136 N/m, if one prong is rigidly attached to the support. This is due to the area connecting the two prongs, which is deformed during the oscillation. This is not only important for the spring constant but also for the dissipation of the TF. Dynamic measurements have shown that applying glue to that area will reduce the quality factor of the TF by a factor of about 2. As expected the contribution of the layer of glue used to attach the TF cannot be neglected either. Not only the type of glue, but also the thickness and the embedding is important. FEM was used to study the influence of each parameter separately, which is experimentally not possible. As expected the layer of glue should be thin and the prong should be embedded a little bit to obtain a high spring constant. These results explain also the huge error in *k* measured during the ex situ experiments. Some tuning forks are probably glued with some small tilt or a small variation in the amount of the used glue.

Simon et al. [21] already performed FEM simulations for a tuning-fork sensor that is rather different from ours, because both prongs are free. Their calculation as well as ours shows that the area linking the two prongs substantially reduces the spring constant of the tuning fork. The qPlus configuration presented here is different from the conventional qPlus configuration used by Omicron. The results cannot be compared directly to the latter configuration. Nevertheless, it is to be expected that the effect of the glue on the spring constant will not be negligible either.

In summary, the combination of experimental techniques and numerical simulation provides insight into the contributions of various parameters to the spring constant of tuning fork sensors used for dynamical force microscopy. This is of major importance whenever quantitative values for the force gradient, the force, or the dissipated power are to be evaluated.
